# Anomalies in Digestion

**Published:** 1877-06

**Authors:** James Blake


### ARTICLE VIII.

### *Anomalies in Digestion.*

BY JAMES BLAKE, M. D.

One of the most curious features connected with the pathology of digestion is the great difference in relative digestibility of different sorts of food for different stomachs. I do not now allude to the differences met with in the capability of some strong stomach to digest all sorts of food, many of which are well known as unwholesome articles of diet to the ordinary every-day stomachs. But we occasionally meet with stomachs which quite upset the lessons obtained through St. Martin's gastric fistula, and which although certainly not strong, yet show a remarkable facility for digesting some of the normally most indigestible substances.

It has seemed to me that at least in some of these cases we have stomachs which bear a certain resemblance in their digestive capabilities to some of the lower animals. Nor is this at all surprising, for as we frequently meet in man structural resemblances to the lower mammalia in certain organs, I see no reason why we should not find functional resemblances as well. This a point in comparative physiology which has not, I believe, attracted the attention it deserves, and these observations are made more for the purpose of calling attention to the subject, in the hope of obtaining additional facts, than for any importance they may possess, although in a practical point of view anything that can throw additional light on some of the curious cases of indigestion we occasionally meet with, will be useful.

One of the most striking instances I have seen of this anomalous digestion, is that of a lady who has what may be called a decidedly weak stomach, a stomach to which grease in all its culinary forms is nearly poisonous; which utterly rebels against pies and cakes, hot bread and bacon, and in fact against most of the articles that are tabooed for weak stomachs; and yet she perfectly digests green cucumbers and green apples. In fact, when suffering from an attack of dyspepsia, and perhaps even vomiting the lightest articles of diet, the first thing that be digested is a laid green apple, and this, too, late in the evening before retiring. The same patient digests raw turnips and carrots better than when when they are cooked. Now, there can be no doubt that there must be something peculiar in the functional action of such a stomach; some peculiarity in the properties of the gastric juice, which enables it to act on these uncooked hard masses of woody fibre and cellular tissue in a manner different from the generality of stomachs, or at least of weak stomachs. If this peculiarity in the functions of the organ is connected with anything abnormal in its anatomy, I am unable to say ; but there is one fact in connection with this case that might excite suspicion that it may be so, viz.: the existence of what may be called esophageal dyspepsia, or a habit of retaining a considerable quantity of food in the esophagus, and regurgitating it almost unchanged after it has been down some hours. Whether this is connected with the presence of a diverticulum, or from a simple enlargement of the esophagus, I am not prepared to say, but in either case we have an approach to the anatomical structure of the stomach found in ruminants, whose normal food is celular and fibrous vegetable tissue.—*Pacific Medical and Surgical Journal.*

